# Occupational risk factors for Parkinson's disease: a case-control study in Japan

**DOI:** 10.1186/1471-2377-11-83

**Published:** 2011-07-07

**Authors:** Keiko Tanaka, Yoshihiro Miyake, Wakaba Fukushima, Satoshi Sasaki, Chikako Kiyohara, Yoshio Tsuboi, Tatsuo Yamada, Tomoko Oeda, Takami Miki, Nobutoshi Kawamura, Nobutaka Sakae, Hidenao Fukuyama, Yoshio Hirota, Masaki Nagai, the Fukuoka Kinki Parkinson's Disease Study Group

**Affiliations:** 1 Department of Preventive Medicine and Public Health, Faculty of Medicine, Fukuoka University, Fukuoka, Japan; 2 Department of Public Health, Osaka City University Graduate School of Medicine, Osaka, Japan; 3 Department of Social and Preventive Epidemiology, School of Public Health, The University of Tokyo, Tokyo, Japan; 4 Department of Preventive Medicine, Graduate School of Medical Sciences, Kyushu University, Fukuoka, Japan; 5 Department of Neurology, Faculty of Medicine, Fukuoka University, Fukuoka, Japan; 6 Department of Neurology, Utano National Hospital, Kyoto, Japan; 7 Department of Geriatric Medicine, Osaka City University Graduate School of Medicine, Osaka, Japan; 8 Department of Neurology, Graduate School of Medical Sciences, Kyushu University, Fukuoka, Japan; 9 Human Brain Research Center, Kyoto University Graduate School of Medicine, Kyoto, Japan; 10 Department of Public Health, Saitama Medical University Faculty of Medicine, Saitama, Japan; 11 Other members of the Study Group are listed in the Appendix

## Abstract

**Background:**

The evidence for associations between occupational factors and the risk of Parkinson's disease (PD) is inconsistent. We assessed the risk of PD associated with various occupational factors in Japan.

**Methods:**

We examined 249 cases within 6 years of onset of PD. Control subjects were 369 inpatients and outpatients without neurodegenerative disease. Information on occupational factors was obtained from a self-administered questionnaire. Relative risks of PD were estimated using odds ratios (ORs) and 95% confidence intervals (CIs) based on logistic regression. Adjustments were made for gender, age, region of residence, educational level, and pack-years of smoking.

**Results:**

Working in a professional or technical occupation tended to be inversely related to the risk of PD: adjusted OR was 0.59 (95% CI: 0.32-1.06, *P *= 0.08). According to a stratified analysis by gender, the decreased risk of PD for persons in professional or technical occupations was statistically significant only for men. Adjusted ORs for a professional or technical occupation among men and women were 0.22 (95% CI: 0.06-0.67) and 0.99 (0.47-2.07), respectively, and significant interaction was observed (*P *= 0.048 for homogeneity of OR). In contrast, risk estimates for protective service occupations and transport or communications were increased, although the results were not statistically significant: adjusted ORs were 2.73 (95% CI: 0.56-14.86) and 1.74 (95% CI: 0.65-4.74), respectively. No statistical significance was seen in data concerning exposure to occupational agents and the risk of PD, although roughly a 2-fold increase in OR was observed for workers exposed to stone or sand.

**Conclusion:**

The results of our study suggest that occupational factors do not play a substantial etiologic role in this population. However, among men, professional or technical occupations may decrease the risk of PD.

## Background

The cause of Parkinson's disease (PD) remains unknown, although a complex interaction among genetic and environmental factors is likely to be involved in the development and progression of the disease [1,2]. The incidence rate was estimated to be 16.9 per 100,000 person-years in one Japanese study [3].

Results of studies of twins suggest that genetic factors are important in early-onset PD cases, whereas environmental factors play a predominant etiologic role in late-onset PD patients [4,5]. Among environmental risk factors, long-term life experiences, such as occupation, may be especially important. Previous occupational studies on PD have provided data on specific environmental risk factors, although consistency in the definition of risk factors is lacking. Some studies have reported increased risks associated with farming [5,7], teaching [8], and health care work [8], and reduced risks with service [9] and transport and communication [10] occupations. However, other studies have not found any association between occupations and the risk of PD [11-13]. Many studies have examined possible relationships between exposure to pesticides [14-22], heavy metals [23,24], and solvents [17,24] and increased risk of PD, and some, but not all, reported positive associations. Thus, epidemiological evidence regarding the association between occupational factors and the risk of PD has been inconsistent.

Occupational studies provide a useful approach to the investigation of environmental exposures as markers of exposure to agents used in the workplace [25], and the effects of job strain and job satisfaction [26]. However, to our knowledge, there has been no epidemiological study on the association between occupational risk factors and PD in Japan. The aim of this multicenter hospital-based case-control study was to examine the associations between occupational factors and the risk of PD among Japanese.

## Methods

### Study subjects

Patients with PD were recruited at three university hospitals and one national hospital in Fukuoka Prefecture, a metropolitan area of Kyushu Island in southern Japan, and in three university hospitals, three national hospitals and one municipal hospital in Osaka, Kyoto, and Wakayama Prefectures, which are part of the Kinki region that is in the midwestern part of the mainland. Eligible cases were patients who were within 6 years of the onset of PD and who had been diagnosed by the collaborating neurologists according to the United Kingdom Parkinson's Disease Society Brain Bank clinical diagnostic criteria (steps 1 and 2) [27]. The neurologists in charge asked eligible PD patients to take part in our case-control study. Of 298 eligible PD patients identified during the period from 1 April 2006 to 31 March 2008, 250 agreed to participate in the study (response rate: 84%).

During the same period, control subjects without a previous diagnosis of a neurodegenerative disease were recruited from departments other than the department of neurology in 3 of the 11 collaborating hospitals, namely, one university hospital in Fukuoka Prefecture, and one university hospital and one national hospital in the Kinki region (department of orthopedic surgery, ophthalmology, otorhinolaryngology, plastic surgery, and oral surgery). Control subjects were not, individually or in larger groups, matched to cases. When a potential control subject was seen as an outpatient or was hospitalized in any of these three hospitals, that individual was asked by an attending doctor or one of our research nurses to participate in our case-control study as a control subject. In the end, from a group of 528 potential control subjects, 372 elected to participate in our study, and 156 refused (response rate: 70%).

One case and 3 control subjects were excluded due to missing data on the factors under investigation, so data for 249 cases and 369 control subjects were ultimately available for analysis. The ethics committees of the 11 collaborating hospitals approved our case-control study (Faculty of Medicine, Fukuoka University; Utano National Hospital; Osaka City University Graduate School of Medicine; Graduate School of Medical Sciences, Kyushu University; Wakayama Medical University; Kyoto University Graduate School of Medicine; Kurume University School of Medicine; Minami-Kyoto National Hospital; Toneyama National Hospital; Kyoto City Hospital; and National Omuta Hospital). Written informed consent was obtained from all subjects.

### Data collection

Participants filled out a set of two self-administered questionnaires and mailed these materials to a data management center or handed them directly to research nurses. Our research technicians completed missing answers and/or corrected illogical data using telephone or direct interviews.

One of the self-administered questionnaires elicited information on gender, age, educational level, smoking habits, occupational history, and exposure to specific occupational agents (metal, wood, asbestos, coal, stone and sand, organic solvents, chalk, pesticides, herbicides and fungicides).

The second questionnaire was a validated self-administered diet history questionnaire, however dietary data were not used in the current study.

### Exposure assessment

In Japan, because most workers are in traditional lifetime employment systems, relatively few experience plural occupations during their lifetime. Therefore, the employment data used in this study focused on the type of job held for the longest period of time during the subject's working life. The mean duration of the job held for the longest time in the study population was 24.8 years. Occupational exposure to agents was defined as being present if the subject reported exposure for 10 or more hours per week for more than 1 year.

### Statistical analysis

Jobs held for the longest period of time were coded using the Japanese Standard Occupational Classification and stratified into 11 major groups (professional or technical; managerial or official; clerical or related occupation; sales; service; protective service; farming, fishing or forestry; transport or communication; production; materials handling; and construction or extraction). Multiple logistic regression analysis was used to estimate adjusted odds ratios (ORs) and 95% confidence intervals (CIs) for PD in relation to occupational factors. Gender, age, region of residence, educational level, and pack-years of smoking were used as confounding variables. The following factors were classified: region of residence (Fukuoka and Kinki), educational level (< 10, 10-12, and ≥ 13 years), and pack-years of smoking (none, 0.1-29.9, and ≥ 30.0). Age was used as a continuous variable. The reference category for all occupational factors was based on a comparison of those exposed to a single agent with all those who were unexposed to that agent, including potential subjects who were exposed to other etiologic factors. The crude and adjusted risk estimates were nearly identical; therefore we reported only adjusted results. All analyses were performed using the SAS software package version 9.2 (SAS Institute, Cary, NC, USA).

## Results

The characteristics of cases and control subjects are summarized in Table 1. Cases and control subjects had a similar gender distribution. Cases were more likely to be older and report never having smoked. Region of residence and educational level were similar in the two groups.

**Table 1:** Characteristics of Parkinson's disease cases and control subjects

Variable	n (%) or Mean (SD)	
	
	Cases, n = 249	Controls, n = 369
Gender (male)	93 (37.4)	141 (38.2)
Age (years)	68.5 (8.6)	66.6 (8.5)
Region of residence		
Kinki	160 (64.3)	215 (58.3)
Fukuoka	89 (35.7)	154 (41.7)
Educational level (years)		
< 10	51 (20.5)	78 (21.1)
10-12	122 (49.0)	171 (46.3)
≥ 13	76 (30.5)	120 (32.5)
Pack-years of smoking		
None	185 (74.3)	222 (60.2)
0.1-29.9	37 (14.9)	65 (17.6)
30.0 ≤	27 (10.8)	82 (22.2)

Table 2 presents adjusted ORs and 95% CIs for PD in relation to the job held for the longest period of time. For the majority of occupational groups, estimates were near the null value. Working in a professional or technical occupation tended to be inversely related to the risk of PD: adjusted OR was 0.59 (95% CI: 0.32-1.06, *P *= 0.08). On the other hand, risk estimates for protective service occupations and transport or communications were increased, although the results were not statistically significant: adjusted ORs were 2.73 (95% CI: 0.56-14.86) and 1.74 (95% CI: 0.65-4.74), respectively.

**Table 2:** Adjusted ORs for Parkinson's disease in relation to occupational groups

Category	n (%)		Adjusted ORs*	95% CI
			
	Cases, n = 249	Controls, n = 369		
Professional or technical	20 (8.0)	45 (12.2)	0.59	0.32-1.06
Manager or official	36 (14.5)	47 (12.7)	1.20	0.69-2.06
Clerical or related occupation	63 (25.3)	76 (20.6)	1.36	0.91-2.04
Sales	19 (7.6)	36 (9.8)	0.87	0.47-1.56
Service	12 (4.8)	22 (6.0)	0.80	0.37-1.67
Protective service	4 (1.6)	3 (0.8)	2.73	0.56-14.86
Farming, fishing, or forestry	11 (4.4)	16 (4.3)	0.95	0.41-2.15
Transport or communications	10 (4.0)	9 (2.4)	1.74	0.65-4.74
Production	39 (15.7)	48 (13.0)	1.11	0.68-1.81
Materials handling	0	2 (0.5)	--	--
Construction or extraction	14 (5.6)	21 (5.7)	1.25	0.59-2.60

OR = odds ratio; CI = confidence interval.

*Adjusted for gender, age, region of residence, educational level, and pack-years of smoking/

Results for occupational exposures are shown in Table 3. There was no statistical significance concerning exposure to any of the occupational agents and the risk of PD, although roughly a 2-fold increase in OR was observed for exposure to stone or sand: adjusted OR = 1.98 (95% CI: 0.39-11.18).

**Table 3:** ORs for Parkinson's disease in relation to exposure to occupational agents

Category	n (%)		Adjusted ORs*	95% CI
			
	Cases, n = 249	Controls, n = 369		
Any	36 (14.5)	61 (16.5)	0.90	0.56-1.43
Metal	6 (2.4)	7 (1.9)	1.26	0.38-4.01
Wood	5 (2.0)	10 (2.7)	0.95	0.28-2.89
Asbestos	1 (0.4)	7 (1.9)	0.23	0.01-1.40
Coal	2 (0.8)	2 (0.5)	1.02	0.12-8.97
Stone or sand	4 (1.6)	3 (0.8)	1.98	0.39-11.18
Solvents	7 (2.8)	12 (3.3)	1.10	0.38-2.95
Chalk	5 (2.0)	6 (1.6)	1.18	0.32-4.18
Pesticides	15 (6.0)	28 (7.6)	0.75	0.37-1.46
Herbicides	12 (4.8)	19 (5.2)	0.87	0.39-1.88
Fungicides	7 (2.8)	12 (3.3)	0.94	0.34-2.47

OR = odds ratio; CI = confidence interval.

*Adjusted for gender, age, region of residence, educational level, and pack-years of smoking.

To examine whether gender or smoking status affected the association between occupations, or exposure to occupational agents, and the risk of PD, we conducted further analyses that were stratified by gender and smoking status. When stratifying the subjects according to gender, a decreased risk of PD in relation to professional or technical occupations was significant only among men. Adjusted ORs for professional or technical occupations among men and women were 0.22 (95% CI: 0.06-0.67) and 0.99 (0.47-2.07), respectively, and significant interaction was observed (*P *= 0.048 for homogeneity of OR). Regarding occupations other than professional or technical occupations and exposure to any of the occupational agents, there were no measurable differences by gender. No significant interactions were observed between any of the exposures under study and smoking status with regard to the risk of PD.

## Discussion

Results from the present case-control study in Japan show a tendency for an inverse association between professional or technical occupations and the risk of PD. No statistically significant relationships between exposure to the occupational agents under study and the risk of PD were observed.

A case-control study in four European centers (Scotland, Sweden, Italy, and Romania) showed a lack of association between professional, technical and managerial occupations and the risk of PD [10]. Also no association between having a professional occupation and the risk of PD was also observed in a case-control study in South Korea [6]. The results of these studies are difficult to compare due to differences in the study populations and the classification of the occupations examined. In the current study, a professional or technical occupation included scientists, teachers, and healthcare workers, such as pharmacists and nurses. In a case-control study in Canada, teaching and healthcare service occupations were associated with an elevated risk of PD; the ORs were 2.5 (95% CI: 1.67-3.75) and 2.07 (95% CI: 1.34-3.20), respectively [8]. On the other hand, in a US case-control study, a null relationship between healthcare or teaching occupations and the risk of PD was observed [13]. Unfortunately, even though information pertaining to subcategories was available, we were not able to analyze professional or technical occupations further because there were too few people in each subcategory to support meaningful analysis.

Possible biological mechanisms that might protect those in professional or technical occupations against PD are unknown. Since occupations classified as professional or technical include a wide range of work activities and potential exposures, identifying specific factors that might be protective is likely to be extremely difficult. Some common lifestyle factors among those holding such positions might be responsible for the decreased risk estimate observed. Alternatively, levels of job strain and decision latitude in relation to occupation may contribute to a decreased risk of PD [26]. In general, greater levels of decision latitude are associated with a better health outcome [26]. Given a positive relationship between professional or technical occupations and decision latitude, the observed inverse association with professional or technical occupations among the subjects of this report might, to some extent, be attributable to greater decision latitude, but data on decision latitude were not available in the current study.

Our data did not show any significant association between farming and exposure to pesticides, herbicides, and fungicides and the risk of PD. Our results are in agreement with previous epidemiological studies that showed no associations between farming or exposure to pesticides, herbicides, or rodenticides and the risk of PD [8-14]; however, they are at variance with the results of other studies showing positive associations between working in agriculture and exposure to pesticides and the risk of PD [6,7,15-19,21,22]. In a meta-analysis based on 19 studies, the combined risk estimate for pesticides in relation to PD was 1.94 (95% CI: 1.49-2.53) [28]. In the present study, since cases and control subjects were both derived from an urban/suburban area and there were small numbers of exposed subjects in each of the exposure categories, detecting statistical significance may be difficult. The term "pesticide" is broad and includes chemicals with various mechanisms of biological action, and information for specific chemicals was not available for the present study. Previous studies have indicated that exposure to specific pesticides, such as parathion and 2,4-Dichlorophenoxyacetic acid, were associated with an increased risk of PD [13,15]. Alternatively, it is possible that the components in the pesticides used in Japan are different from those in other countries.

A particular strength of our study is that since all PD patients were diagnosed by a neurologist according to established criteria, there is little reason to suspect that a misdiagnosis of PD occurred. Also, the response rate among eligible cases was relatively high (84%). Although we took into consideration information on potential confounders, the effects of residual confounding could not be ruled out.

Our study has, however, some important limitations. As our sample size was relatively small, we may have failed to detect associations with various occupational categories or occupational exposures due to a lack of statistical power. In the present study, occupational categories based on the Japanese Standard Occupational Classification were used and the classification is somewhat crude. Additionally, we considered only the longest job held, rather than all occupations held by individuals, and occupational information was based on self-reporting. Moreover, with regard to occupational agents, information on exposure patterns such as frequency or concentration, and exposure modifiers such as work practices, local ventilation systems, or the use of personal protective devices during work, was not available. Consequently, the possibility of inaccurate exposure data and resultant misclassification bias should be considered when interpreting our findings. Another problem with retrospective case-control studies is recall bias, as patients in a case-control study may attribute greater significance to past events and perceived environmental exposure than the control subjects. Since the recruitment of case participants was conducted at 11 collaborating hospitals, whereas control subjects were selected from one university hospital in Fukuoka Prefecture, and one university hospital and one national hospital in the Kinki region, it is possible that the control subjects did not entirely represent the population from which cases were drawn.

## Conclusions

The present study showed the lack of significant associations between occupational factors and the risk of PD. Our findings suggest that occupational factors did not play a substantial etiologic role in this population. However, since our study had several limitations, such as the small number of study subjects, the possibility of recall bias, and the possibility of inaccurate exposure data, our results are not conclusive. Further studies with larger study populations and more accurate measures of exposure are needed to more clearly identify etiologic factors.

## Appendix

Other members of the Fukuoka Kinki Parkinson's Disease Study Group are as follows: Yasuhiko Baba and Tomonori Kobayashi (Department of Neurology, Faculty of Medicine, Fukuoka University); Hideyuki Sawada, Eiji Mizuta, and Nagako Murase (Clinical Research Institute and Department of Neurology, Utano National Hospital); Tsuyoshi Tsutada and Hiroyuki Shimada (Department of Geriatrics and Neurology, Osaka City University Graduate School of Medicine); Jun-ichi Kira (Department of Neurology, Neurological Institute, Graduate School of Medical Sciences, Kyushu University); Tameko Kihira and Tomoyoshi Kondo (Department of Neurology, Wakayama Medical University); Hidekazu Tomimoto (Department of Neurology, Kyoto University Graduate School of Medicine); Takayuki Taniwaki (Division of Respirology, Neurology, and Rheumatology, Department of Medicine, Kurume University School of Medicine); Hiroshi Sugiyama and Sonoyo Yoshida (Department of Neurology, Minami-Kyoto National Hospital); Harutoshi Fujimura and Tomoko Saito (Department of Neurology, Toneyama National Hospital); Kyoko Saida and Junko Fujitake (Department of Neurology, Kyoto City Hospital); Naoki Fujii (Department of Neurology, Neuro-Muscular Center, National Omuta Hospital); Masatoshi Naito and Jun Arimizu (Department of Orthopaedic Surgery, Faculty of Medicine, Fukuoka University); Takashi Nakagawa, Hirofumi Harada, and Takayuki Sueta (Department of Otorhinolaryngology, Faculty of Medicine, Fukuoka University); Toshihiro Kikuta and George Umemoto (Department of Oral and Maxillofacial Surgery, Faculty of Medicine, Fukuoka University); Eiichi Uchio and Hironori Migita (Department of Ophthalmology, Faculty of Medicine, Fukuoka University); Kenichi Kazuki, Yoichi Ito, and Hiroyoshi Iwaki (Department of Orthopaedic Surgery, Osaka City University Graduate School of Medicine); Kunihiko Siraki and Shinsuke Ataka (Department of Ophthalmology and Visual Sciences, Osaka City University Graduate School of Medicine); Hideo Yamane and Rie Tochino (Department of Otolaryngology and Head and Neck Surgery, Osaka City University Graduate School of Medicine); Teruichi Harada (Department of Plastic and Reconstructive Surgery, Osaka City University Graduate School of Medicine); and Yasushi Iwashita, Motoyuki Shimizu, Kenji Seki, and Keiji Ando (Department of Orthopedic Surgery, Utano National Hospital).

## Abbreviations

CI: confidence interval; OR: odds ratio; PD: Parkinson's disease.

## Competing interests

The authors declare that they have no competing interests.

## Authors' contributions

KT contributed to the study design, data collection, data management, statistical analysis, data interpretation, and writing of the manuscript. YM contributed to the study design, data collection, overall management, and manuscript editing. WF contributed to the study design, data collection, and data management. SS and CK contributed to the study design. YT, TY, TO, TM, NK, NS and HF contributed to the outcome definition and case recruitment. YH and MN contributed to the supervision of the design and execution of the study. Authors listed in the Appendix contributed to case or control subject recruitment. All authors contribute to the preparation of the manuscript and approved the final version submitted for publication.

## Pre-publication history

The pre-publication history for this paper can be accessed here:

http://www.biomedcentral.com/1471-2377/11/83/prepub
